# Combined thermal ablation and liposomal granulocyte-macrophage colony stimulation factor increases immune cell trafficking in a small animal tumor model

**DOI:** 10.1371/journal.pone.0293141

**Published:** 2023-10-26

**Authors:** Marwan Moussa, Md. Raihan Chowdhury, David Mwin, Mohamed Fatih, Gokul Selveraj, Ahmed Abdelmonem, Mohamed Farghaly, Qianhui Dou, Nina Filipczak, Tatyana Levchenko, Vladimir P. Torchilin, Vassiliki Boussiotis, S. Nahum Goldberg, Muneeb Ahmed

**Affiliations:** 1 The Laboratory for Minimally Invasive Tumor Therapies, Department of Radiology, Beth Israel Deaconess Medical Center/Harvard Medical School, Boston, Massachusetts, United States of America; 2 Department of Pharmaceutical Sciences, Northeastern University, Boston, Massachusetts, United States of America; 3 Department of Hemotolgy and Oncology, Beth Israel Deaconess Medical Center/Harvard Medical School, Boston, Massachusetts, United States of America; 4 Department of Radiology, Hadassah Hebrew University Medical Center, Jerusalem, Israel; University of Illinois, UNITED STATES

## Abstract

**Purpose:**

To characterize intratumoral immune cell trafficking in ablated and synchronous tumors following combined radiofrequency ablation (RFA) and systemic liposomal granulocyte-macrophage colony stimulation factor (lip-GM-CSF).

**Methods:**

Phase I, 72 rats with single subcutaneous R3230 adenocarcinoma were randomized to 6 groups: a) sham; b&c) free or liposomal GM-CSF alone; d) RFA alone; or e&f) combined with blank liposomes or lip-GM-CSF. Animals were sacrificed 3 and 7 days post-RFA. Outcomes included immunohistochemistry of dendritic cells (DCs), M1 and M2 macrophages, T-helper cells (Th1) (CD4^+^), cytotoxic T- lymphocytes (CTL) (CD8^+^), T-regulator cells (T-reg) (FoxP3^+^) and Fas Ligand activated CTLs (Fas-L^+^) in the periablational rim and untreated index tumor. M1/M2, CD4^+^/CD8^+^ and CD8^+^/FoxP3^+^ ratios were calculated. Phase II, 40 rats with double tumors were randomized to 4 groups: a) sham, b) RFA, c) RFA-BL and d) RFA-lip-GM-CSF. Synchronous untreated tumors collected at 7d were analyzed similarly.

**Results:**

RFA-lip-GMCSF increased periablational M1, CTL and CD8^+^/FoxP3^+^ ratio at 3 and 7d, and activated CTLs 7d post-RFA (p<0.05). RFA-lip-GMSCF also increased M2, T-reg, and reduced CD4^+^/CD8^+^ 3 and 7d post-RFA respectively (p<0.05). In untreated index tumor, RFA-lip-GMCSF improved DCs, M1, CTLs and activated CTL 7d post-RFA (p<0.05). Furthermore, RFA-lip-GMSCF increased M2 at 3 and 7d, and T-reg 7d post-RFA (p<0.05). In synchronous tumors, RFA-BL and RFA-lip-GM-CSF improved DC, Th1 and CTL infiltration 7d post-RFA.

**Conclusion:**

Systemic liposomal GM-CSF combined with RFA improves intratumoral immune cell trafficking, specifically populations initiating (DC, M1) and executing (CTL, FasL^+^) anti-tumor immunity. Moreover, liposomes influence synchronous untreated metastases increasing Th1, CTL and DCs infiltration.

## Introduction

Image-guided, minimally-invasive thermal tumor ablation using radiofrequency (RFA) or microwave (MWA) is now commonly used as a standard of care treatment for a wide range of focal tumors (2–5 cm) most notably primary and metastatic (e.g. from primary colorectal, neuroendocrine and breast cancers) cancer in the liver, but also lung, kidney, bone, and other sites [1–12]. Moreover, thermal ablation is increasingly used to treat oligometastatic tumors and/or isolated tumor progression in the setting of more diffuse metastatic disease [3, 10–13**]**. However, despite successful locoregional control, most patients with metastatic disease will ultimately succumb to their multifocal progression and resistance to systemic lines of therapy.

Beyond inducing immediate cancer cell death from high-temperature heating, thermal ablation also induces a series of secondary reactions including increased periablational and systemic expression of immune-driving cytokines (i.e., IL-6 and IL-10) and periablational markers of inflammation such as heat shock proteins (linked to promotion of antigen presenting cells) [14–18]. Additionally, increased periablational and intratumoral trafficking of various antigen-presenting cell populations and effector cytotoxic T-cells have been observed in both experimental and clinical studied [19]. Moreover, some clinical studies have reported increased responses to tumor-associated antigens in circulating immune cell populations in patients who have undergone thermal ablation for primary liver cancer [20]. Thus, given these findings, there is interest in using local thermal ablation to stimulate secondary anti-tumor immunity-related treatment responses in non-ablated distant tumors (i.e., ‘abscopal effects’).

Despite the rapid rise in the clinical use of systemic immunotherapies such as immune checkpoint inhibitors (ICIs) for a wide range of cancers, there are many tumor types that do not respond robustly to systemic immunotherapy, usually due to an ‘immune-cold’ state from insufficient effective intratumoral immune cell populations or lack of antigen availability from tumor cell paucity. While combinations of systemic immunotherapies to multiple immune targets have increased treatment response rates for metastatic renal cell and melanoma cancers, these are associated with significantly higher rates of treatment-related toxicities [21]–highlighting the continued a need for better combination approaches. Initial studies have further demonstrated that thermal ablation can be successfully combined with adjuvant immunotherapies, such as systemic ICIs or a direct intratumoral immunostimulant injection, to reduce treated tumor growth and induce anti-tumor immunity to tumor cell re-inoculation [22, 23]. Yet, these approaches still have similar limitations of the known systemic side effects from intravenous and oral agents and variable intratumoral delivery from direct injection.

One opportunity to overcome current limitations with combined ablation-immunotherapy approaches is to optimize immunomodulation of the tumor microenvironment by aligning ablation-induced effects with targeted drug delivery to the ablation zone–thereby reducing systemic effects [24, 25]. Serendipitously, thermal ablation induces several local changes that have the potential to foster anti-tumor immunity, including inducing release of tumor antigens following tumor cell death [26] and simultaneously recruiting periablational trafficking of several inactivated antigen-presenting cell populations [27]. These two spatially and temporally-reproducible phenomena could create a favorable milieu for immunomodulation of the tumor microenvironment after thermal ablation–with antigen-presenting cell activation using adjuvant targeted drug immunomodulation.

We have previously demonstrated that nanocarriers (e.g., liposomes and micelles) can be used to effectively delivery targeted therapies (i.e., chemotherapies, siRNA, and small molecule drugs) selectively to the periablational rim in order to directly modulate both hyperthermia-induced local reactions, and downstream effects on distant sites of tumor [24, 25, 28, 29]. Drug-loaded liposomes are preferentially delivered to the periablational rim where increased vascular permeability and vascular dilation occurs; and this matches to the same spatial zone in which increased antigen-presenting cell (i.e., dendritic cells and macrophages) trafficking occurs [30]. In the current study, we exploit this phenomena by liposomal-loading of granulocyte-macrophage colony stimulation factor (GM-CSF), which has a core function of inducing maturation and trafficking of dendritic cells, professional antigen-presenting cells, and M1 pro-inflammatory macrophages. GM-CSF is a key molecule of interest for this reason, and pivotal to several tumor vaccine strategies and combined with radiation [31]. Nevertheless, this drug has been previously limited in use due to difficulties with delivery to the target area. As such, this particular combination that aligns optimal drug delivery and thermal ablation effects with the necessary activation of immune cells has the potential to augment ablation-induced anti-tumor immunity over other reported combination therapies. Accordingly, the purpose of this study is to evaluate the combination of adjuvant liposomal GM-CSF (lip-GM-CSF) following thermal tumor ablation (using RFA) on the tumor immune microenvironment (TME) in local and distant tumor in a rat breast cancer model–with specific attention regarding its effect upon antigen-presenting cell trafficking and maturation (dendritic cells and macrophages) and their downstream effector cell populations (i.e., cytotoxic T cell lymphocytes).

## Materials and methods

### Overview of experimental design

This study was carried out in strict accordance with the recommendations in the Guide for the Care and Use of Laboratory Animals of the National Institutes of Health. The protocol was approved by the Committee on the Ethics of Animal Experiments of Beth Israel Deaconess Medical Center (Protocol Number: 025–2021). All animal procedures were performed under isoflurane anesthesia, and all efforts were made to minimize suffering. The experiment was divided into two phases.

#### Phase I: Effect of combining thermal ablation with liposomal GM-CSF on local ablated tumor microenvironment

A solitary rat R3230 breast adenocarcinoma tumor was implanted orthotopically to simulate an isolated primary adenocarcinoma undergoing incomplete ablation. At a mean diameter of 6–7 mm, tumors were measured daily until they reached a target mean diameter of 11-12mm. Animals were then randomly assigned into six treatment groups (total n = 72): 1) sham; probe placement without applicator activation, 2) free GM-CSF (fr-GM-CSF); intra-peritoneal (IP) injection of free GM-CSF, 3) liposomal GM-CSF (lip-GM-CSF); IP injection of liposomal GM-CSF, 4) Radiofrequency ablation alone (RFA); 1 cm active tip, 21g electrode, energy titrated to maintain a tip temperature of 70°C for 5 min [R], 5) RFA combined with (blank) liposomes, administered (IP) at the time of RFA (RFA-BL), and 6) RFA with liposomal GM-CSF (RFA-lip-GM-CSF). Animals were sacrificed at 3d and 7d following treatment in all groups. Tumor growth analysis (tumor size and growth curve analysis) was performed. Immunohistochemistry of immune cells in tumor microenvironment at the periablational zone and at a distance of two high power field (hpf) away from periablational zone (‘untreated index tumor’) were analyzed. Tumor-associated macrophages (TAMs) were assessed, including CD68^+^ pro-inflammatory (M1) and CD163^+^ anti-inflammatory (M2) macrophage sub-types. Tumor-infiltrating lymphocytes (TILs) were also assessed, including CD4^+^ T-helper cells (Th1), CD8^+^ cytotoxic T-cells (CTLs), FoxP3^+^ T-regulatory cells (T-reg), and activated FasL^+^ CTLs. CD11c^+^ Dendritic cells (DCs) were reported as number of positive cells per sample, due to low number per sample. Furthermore, M1/M2, CD4/CD8, and CD8/FoxP3 ratios in the periablational zone and in untreated index tumor (two high power fields away) were calculated.

#### Phase II: Effect of combining thermal ablation with liposomal GM-CSF on the distant non-ablated tumor microenvironment

A double tumor model implanting two R3230 subcutaneously on contralateral breasts was used, simulating a synchronous metastasis scenario in which an index tumor is treated with incomplete ablation. Informed by the results of phase I, 40 animals were randomized into 4 treatment groups: 1) sham, 2) RFA alone; 3) RFA-BL, and 4) RFA-lip-GM-CSF. Endpoints described above for Phase I were used to analyze immune cell infiltration in the distant second tumor microenvironment.

### Animal models

Female Fischer 344 (F344) rats (14–16 weeks old, 150± 20g) were obtained from Charles River Laboratories, Wilmington, MA, USA. All of the pathogen-free rats were housed under natural light/dark conditions (room temperature: 25± 2°C, relative humidity: 60±10%) for at least 1 week before the experiments in animal facilities with free access to food and water. Animals were cared for and handled according to the Institutional Animal Care Committees of Beth Israel Deaconess Medical Center, Boston, MA.

The experiments and procedures were performed by anesthetizing animals using isoflurane according to protocols. Animals were sacrificed with an overdose of carbon dioxide (Smartbox^™^ CO_2_ Chamber System, EZ systems, Palmer, PA). A rat breast adenocarcinoma cells R3230 (strain: F344) were cultured using Roswell Park Memorial Institute (RPMI-1640) medium supplemented with 10% fetal bovine serum (FBS) and 1% Antibiotic-Antimycotic (AA) and sub-cultured every 3^rd^ d to desire number of plates. A fully (>90%) confluence plate of cells was used to implant in a single animal subcutaneously in the mammary fat pad of F344 rats. Cells preparation, implantation and evaluation techniques were performed by following previously described literature [32, 33]. Briefly, while under anesthesia, the implanted sites were shaved, followed by a slow injection of 0.25–0.3 mL of cell’s suspension (2 x 10^6^ cells) into the mammary fat pad of each rat using an 18-gauge needle. Tumor growth was monitored every 1-2d until reached at 6–7 mm in diameter to include animals in studies, after that they were measured every day until 11–13 mm, at which point they were randomized to different groups for ablation treatment.

### Radiofrequency ablation

The animals were ablated by conventional monopolar method using a 500-KHz RF ablation generator (Model RFG-1B, Cosman Medical Inc., Burlington, Massachusetts). At first, the tip (1-cm) of a 21-gauge electrically insulated electrode (SMK electrode; Radionics Inc.,) was inserted at the center of the tumor. RF ablation was applied for 5 minutes with generator output titrated to retain a designated tip temperature of 70°C ± 2 mean with a range of 48–160 mA. The animal’s back was shaved properly to complete the RF circuit easily. A water-soluble ultrasound gel was also applied on the shaved skin to ensure proper conduction with the metallic grounding pad (Radionics Inc).

### Liposomal GM-CSF formulation and administration

Freshly prepared liposomal GM-CSF and its placebo formulation were provided by the Department of Pharmaceutical Sciences of Northeastern University (Boston, Massachusetts). The formulations were prepared using the following method. Briefly, a lipid film from mixture of hydrogenated soy phosphatidylcholine, cholesterol and polyethyleneglycol phosphatidylethanolamine (PEG2000-PE) (57.25:37.57:5.18 mol%, respectively) in chloroform was formed in a round-bottomed flask by solvent removal on a rotary evaporator and hydrated with GM-CSF solution in phosphate buffered saline, pH 7.4 (the final concentration 14 μg/ml). Suspension was vortexed for a few seconds and sonicated with water bath. After three freeze thaw cycles liposomes were extruded through 200 nm filters. Dialyzed overnight (membrane 100 kDa) liposomes were used for injection after protein determination with Micro BCA kit. Th liposomes were stored at 4°C temperature in a lightproof closed container until administration. The RFA-lip-GM-CSF rats were injected with a single intraperitoneal (IP) dose of 50μg/kg (ie, 0.5 mL of lip-GM-CSF solution only once, 15 minutes after RF ablation). The method of administration, time gap between ablation & administration and techniques of preparation of lip-GM-CSF were optimized according to previously published papers by our group [34, 35]. Briefly, in the same animal model the effects of the following variables on tumor necrosis and systemic toxicity were investigated. Liposome size, time of administration in relation to ablation and liposomal agents. Parameters with greatest tumor necrosis and least systemic toxicity were selected.

### Immunohistochemistry

The sections made from paraffin embedded tumors were immunohistochemically stained to evaluate 1) TAMs, including CD68^+^ pro-inflammatory (M1) and CD163^+^ anti-inflammatory (M2) macrophage sub-types 2) TILs, including CD4^+^ T-helper cells (Th1), CD8^+^ cytotoxic T-cells (CTLs), FoxP3^+^ T-regulatory cells (T-reg),and activated FasL^+^ CTLs and 3) CD11c^+^ Dendritic cells (DCs)were also performed in the periablational rim and untreated index tumor, for phase I. Untreated index tumor was defined as two high power fields away from the ablation zone. For phase II, CD68^+^, CD163^+^, CD4^+^, CD8^+^, FoxP3^+^ and CD11C^+^ were stained. To ensure the uniformity of staining, the immunohistochemical examination whenever for direct comparison was repeated with all relevant slides stained at the same time. Briefly, the sections were incubated for 1 hour with the primary antibody. The slides were washed with TBS and followed by a 30 minute incubation with a biotinylated secondary antibody (S1 Table in S1 File). The slides were washed with TBS and treated with diaminobenzidine (DAB, Abcam, Cambridge) chromogen for 2–5 minutes. Finally, slides were counterstained by hematoxylin and dehydrated, and mounted for microscopic observation. The experiments were performed by individuals with 3–15 years of experience in performing immunohistochemistry (D.M, M.A, M.M, G.S, and M.R.C.). The obtained data were verified by the senior author (M.A.).

### Immunohistochemistry ImageJ analysis

Using the image J software, the IHC stained image was opened and the staining type was selected. Color deconvolution was then performed, resulting in the original image being separated into three single-colored images. The second color image was then chosen and its threshold was adjusted by selecting pixels within the red box in the Threshold window. The selected area was modified and applied to create a binary image using the bars (0–255). The watershed function was applied to separate cells that were close together, though this method may not be accurate for crowded cells. Finally, the Analyze Particles function was used, setting a minimum size to remove small dots selected within the threshold that are not cells.

### Statistical analysis

All data were expressed as means ± standard deviations (SD). The mean tumor sizes at selected time point (Day 0 and at the time of sacrifice), and immunohistochemical determination were compared using analysis of variance (ANOVA). Additional post-hoc analysis was performed with paired, two-tailed Student’s *t*-test, if and only if, the analysis of variance achieved statistical significance. The p value was considered as significant in case of < 0.05.

## Results

### Combining thermal ablation with liposomal GM-CSF improves TAM and DC infiltration in the periablational zone and untreated index tumor

**Fig 1:**
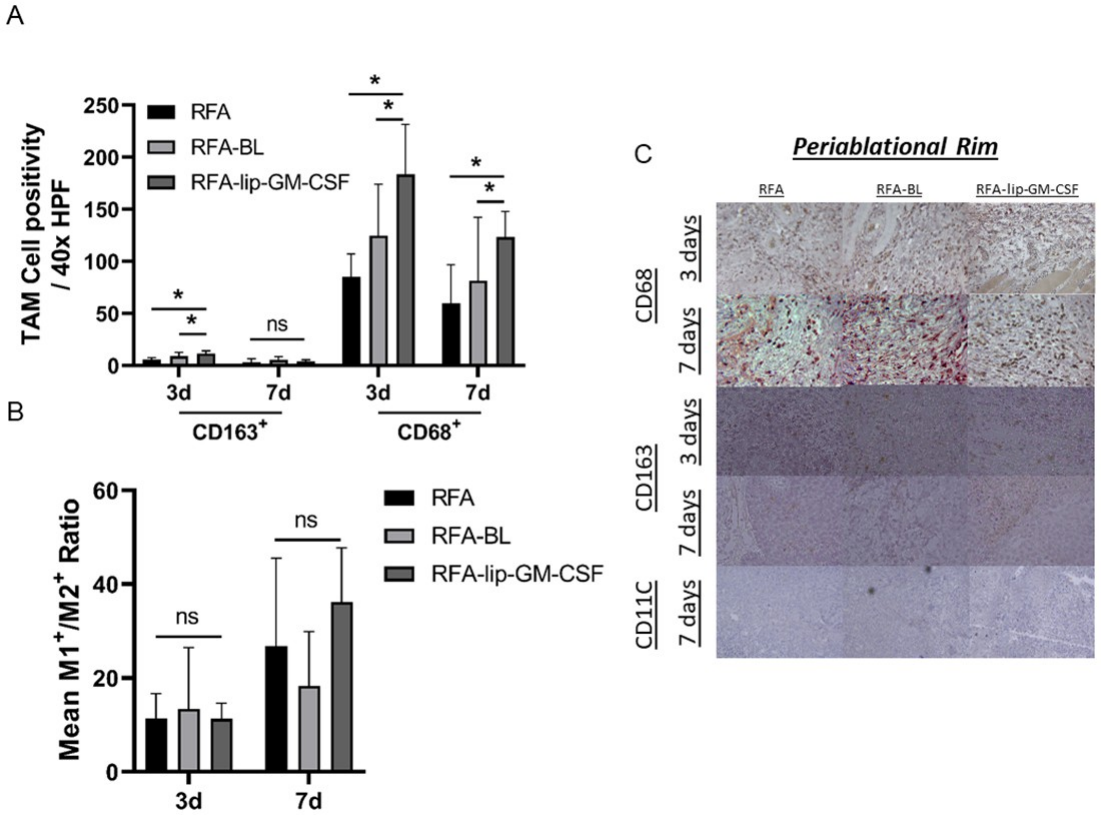
Effect of liposomal GM-CSF and RFA on antigen presenting cells in R3230 rat model on 3 and 7 days post ablation in the periablational rim. (A) At 3 days post treatment TAM (M2: CD163^+^ and M1: CD68^+^) infiltration in the tumor periablational in RFA-lip-GM-CSF group was higher than other groups, but not at 7 days. M1^+^/M2^+^ ratio in the tumor periablational rim (B) demonstrated no differences between groups. Immunohistochemistry representative images of infiltrating APCs in the periablational rim (C). *, p< 0.05, RFA-lip-GM-CSF vs all groups, **, p< 0.05, RFA-lip-GM-CSF vs all groups, except RFA-BL, ns, not significant.

A 2.2-fold and 2.1-fold increase in CD68^+^ M1 macrophages were detected in the periablational rim at 3d and 7d after treatment, respectively, with RF-lip-GM-CSF compared to Radiofrequency ablation alone (RFA) (p<0.05). There were also significantly increased CD68^+^ M1 macrophages compared to RFA-BL at 3d and 7d (p<0.05) (Fig 1A and 1C). Similarly, RFA-lip-GM-CSF resulted in a 2.1-fold increase in CD163^+^ M2 macrophages compared to RFA at 3d (p<0.05). Yet, at 7d, M2 macrophage levels had equilibrated across all treatment groups, in the periablational zone. (Fig 1A and 1C) (Table 1).

**Table 1:** Effect of combining thermal ablation and liposomal GM-CSF on periablational rim and untreated index tumor infiltration of tumor associated macrophages and dendritic cells.

*Periablational rim*			Sham	fr-GM-CSF	lip-GM-CSF	RFA	RFA-BL	RFA-lip-GM-CSF	P value
	Cellular infiltrates (PC/hpf±SD)								
	CD68^+^	3D	-	-	-	85.2±21.9	124.6±49.5	183.5±47.8	*
		7D	-	-	-	59.6±37.1	81.5±60.9	123.3±24.6	*
	CD163^+^	3D	-	-	-	5.7±2.0	9.0±3.8	11.6±2.4	*
		7D	-	-	-	3.3±3.2	5.6±2.8	3.9±1.6	ns
	M1:M2 ratio								
	M1:M2	3D	-	-	-	11.4 ±5.3	13.4 ±13.1	11.3 ±3.3	ns
		7D	-	-	-	26.8±18.7	18.3±11.6	36.2±11.5	ns
*Untreated index tumor*									
	Cellular infiltrates (PC/hpf±SD)								
	CD68^+^	3D	103.2±36.9	119.6±14.1	78.3±19.2	116.3±43.0	160.0±29.1	321.3±61.3	*
		7D	108.0±88.8	156.2±109.1	167.1±37.8	195.4±23.1	326.1±47.1	297.2±41.0	**
	CD163^+^	3D	1.2±0.4	2.6±1.2	5.5±0.4	7.6±2.0	10.6±1.7	18.6±4.0	*
		7D	0.8±0.2	1.1±0.4	2.8±0.8	4.1±2.9	4.8±2.2	9.2±2.9	*
	CD11c^+^		-	-	-	-	-	-	
		7D	2.3±0.6	1.0±1.0	1.3±2.3	8.9±10.3	8.4±7.3	51.6±47.9	*
	TAM ratio								
	M1:M2	3D	80.0±33.3	53.3 ±21.4	29.2±5.4	26.6±3.6	31.6±7.4	15.1±11.5	*
		7D	139.3±99.5	134.7±72.3	62.8±18.5	83.8±66.9	82.8±37.9	32.0±8.1	ns

Abbreviations: fr-GM-CSF: Free form GM-CSF, lip-GM-CSF: Liposomal GM-CSF, RFA: Radiofrequency ablation, BL: Blank Liposomes, TAM: Tumor associated Macrophage, ns: no significant difference.

P value < 0.05.

* RFA-lip-GM-CSF vs all groups,

** RFA-lip-GM-CSF vs all groups, except RFA-BL.

**Fig 2:**
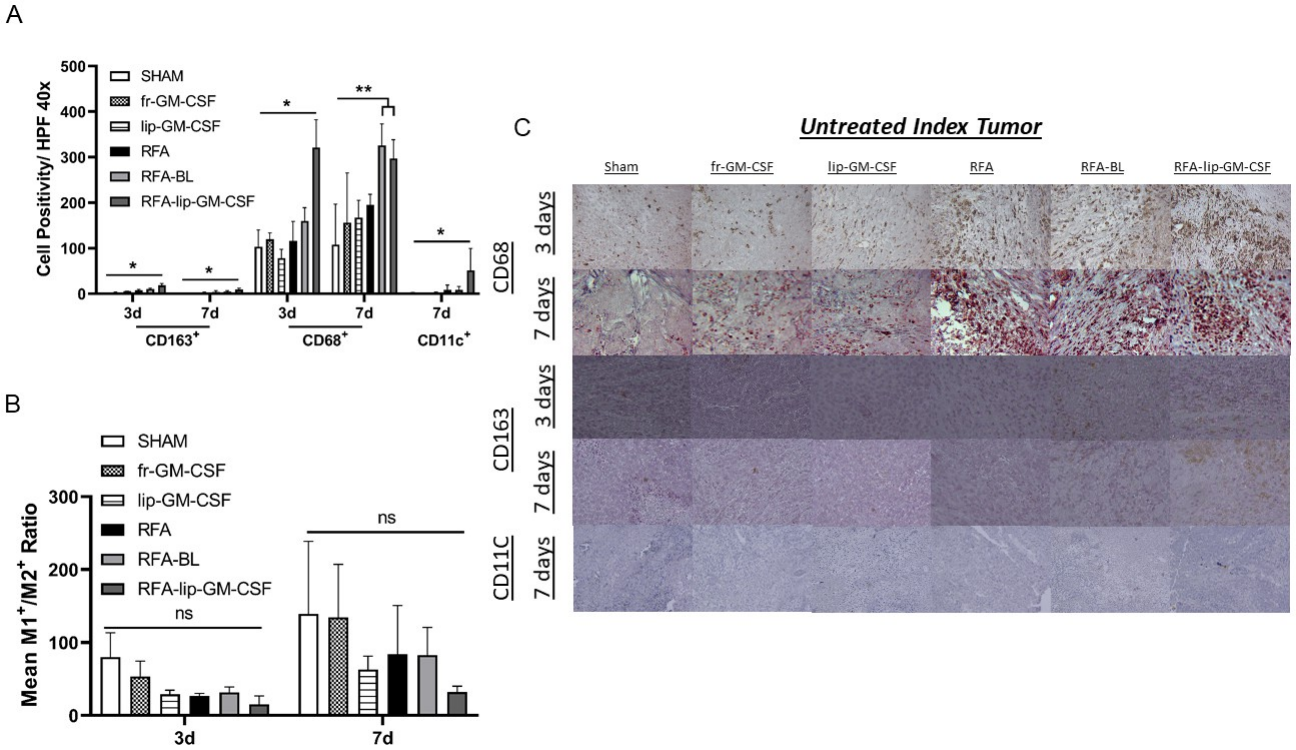
Effect of liposomal GM-CSF and RFA on antigen presenting cells in R3230 rat model on 3 and 7 days post ablation in untreated index tumor. RFA-lip-GM-CSF group expression of TAMs and DCs (A) was higher at 3 and 7 days post treatment, and 7 days only, respectively, when comparing RFA-lip-GM-CSF to all other groups. M1^+^/M2^+^ ratio (B) demonstrated no differences between groups. Immunohistochemistry representative images of infiltrating APCs in untreated index tumor (C). *, p< 0.05, RFA-lip-GM-CSF vs all groups, **, p< 0.05, RFA-lip-GM-CSF vs all groups, except RFA-BL, ns, not significant.

In non-ablated index tumor, a similar pattern was observed two high power-fields away from the ablation. RFA-lip-GM-CSF increased M1 macrophage infiltration at 3d (2.8-fold RFA) and at 7d (1.5-fold RFA) compared to RFA-BL (3d only), RFA alone, liposomal GM-CSF alone, free GM-CSF and sham (p<0.05) (Fig 2A and 2C). RFA-lip-GM-CSF increased CD163^+^ M2 macrophages in untreated index tumor compared to all groups (p<0.05), at 3d and 7d. Finally, CD11c^+^ staining demonstrated a 5.7-fold increase of dendritic cell infiltration at 7 days in RFA-lip-GM-CSF compared to RFA, and was greater than all other treatment groups (p<0.05) (Fig 2A and 2C). CD11c+ was not detected at 3 days (Table 1).

### Combination RFA-lip-GM-CSF increases the M1/M2 ratio 2 in untreated index tumor

RFA-lip-GM-CSF shifted the macrophage cell population toward M2 dominance (M1:M2 ratios) in the untreated index tumor at 3d when compared to all other groups (p<0.05). By 7 days post ablation, findings had reverted to control baseline so that no difference in M1/M2 ratios was noted between groups (Fig 1B) (Table 1).

### Combined RFA-lip-GM-CSF improves TIL infiltration in the periablational rim and untreated index tumor

**Fig 3:**
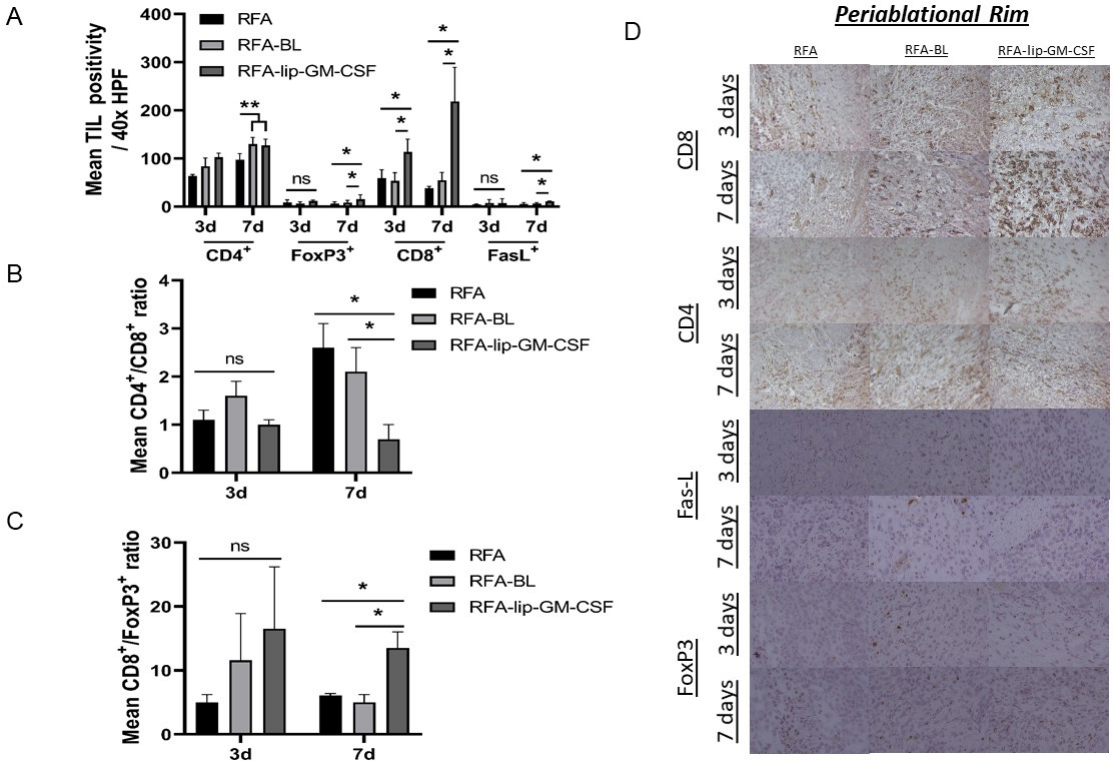
Effect of liposomal GM-CSF and RFA on tumor infiltrating lymphocytes in R3230 rat model on 3 and 7 days post ablation in the periablational rim. In the periablational rim (A) TILs expression (CD4^+^: Th1, FoxP3^+^:Treg, CD8^+^:CTL and FasL^+^: Activated CTL) increased in RFA-lip-GM-CSF group compared to other groups at 7 days (7d), but not at 3 days (3d). CD4^+^/CD8^+^ ratio in the periablational rim (B) decreased in in RFA-lip-GM-CSF group compared to other groups at 7 days, but not at 3 days. For CD8^+^/FoxP3^+^ ratio, in the periablational rim (C) RFA-lip-GM-CSF group demonstrated a higher ratio than other groups at 7 days, but not at 3 days. Immunohistochemistry representative images of infiltrating TILs in the periablational rim (C). *, p< 0.05, RFA-lip-GM-CSF vs all groups, **, p< 0.05, RFA-lip-GM-CSF vs all groups, except RFA-BL, ˟˟, p< 0.05, RFA-lip-GM-CSF, RFA-BL, RFA alone vs. lip-GM-CSF alone, fr-GM-CSF and sham, ns, not significant.

In the periablational zone, RFA-lip-GM-CSF increased CD8^+^ CTLs by 1.9-fold at 3d and 5.6-fold at 7d compared to RFA and RFA-BL arms (p<0.05). RFA-lip-GM-CSF also increased FasL^+^ CTL (a marker of CTL cytotoxicity) in the periablational zone at 7d compared to other treatment groups (p<0.05). No significant differences in FasL^+^ cells were detected at 3d. (Fig 3A and 3D). RFA-lip-GM-CSF also increased CD4^+^ T helper cells (Th1) in the periablational zone compared to RFA at 3d and 7d (p<0.05). By contrast, RFA-BL increased CD4+ Th1 cells compared at 7 days only, as compared to RFA. (Fig 3A and 3D). Finally, RFA-lip-GM-CSF also increased periablational zone FoxP3^+^ T-reg cell infiltration at 7d compared to all groups (p<0.05). No significant differences were observed at 3d (Fig 3A and 3D) (Table 2).

**Table 2:** Effect of combining thermal ablation and liposomal GM-CSF on periablational rim and untreated index tumor infiltration of tumor infiltrating lymphocytes.

*Periablational rim*			Sham	fr-GM-CSF	lip-GM-CSF	RFA	RFA-BL	RFA-lip-GM-CSF	P value
	Cellular infiltrates (PC/hpf±SD)								
	CD8^+^	3D	-	-	-	59.3±17.4	53.5±17.0	113.4±26.7	*
		7D	-	-	-	38.5±3.9	54.6±16.1	218.4±70.6	*
	CD4^+^	3D	-	-	-	63.7±3.1	83.8±17.3	102.5±8.5	**
		7D	-	-	-	97.1±13.1	129.9±13.6	127.1±13.2	**
	Fas-L^+^	3D	-	-	-	4.9±0.5	7.9±6.7	7.2±9.4	ns
		7D	-	-	-	5.5±3.9	6.5±1.1	11.2±0.2	*
	FoxP3^+^	3D	-	-	-	8.9±5.2	6.3±3.6	11.9±1.9	ns
		7D	-	-	-	6.3±3.5	8.5±4.5	15.6±8.8	*
	TIL ratios								
	CD4^+^/CD8^+^	3D	-	-	-	1.1±0.2	1.6±0.3	1.0±0.1	ns
		7D	-	-	-	2.6±0.5	2.1±0.5	0.7±0.3	*
	CD8^+^/FoxP3^+^	3D	-	-	-	5.0±1.2	11.6±7.3	16.5±9.7	ns
		7D	-	-	-	6.1±0.3	5.0±1.2	13.5±2.5	*
*Untreated index tumor*									
	Cellular infiltrates (PC/hpf±SD)								
	CD8^+^	3D	58±7.1	85.5±16.3	65.6±21.5	67.2±13.8	46.0±14.9	71.3±26.8	ns
		7D	25.8±2.1	27.5±0.7	38.1±5.8	63.5±3.7	106.6±27.2	123.9±33.0	*
	CD4^+^	3D	25.6±5.1	55±9.3	40.7±2.4	32.8±1.8	25.6±3.2	31.2±13.5	****
		7D	69.1±18.7	102±13.8	131.2±17.9	89.9±15.1	127.6±8.6	135.0±23.1	˟
	Fas-L^+^	3D	3.1 ±1.1	5.8 ±1.2	10.6 ±1.3	3.2 ±2.1	4.7±2.1	6.5 ±6.4	ns
		7D	2.8±1.8	7.5±1.5	7.1±4.8	6.8±3.5	7.8±1.4	13.5±1.2	*
	FoxP3^+^	3D	3.1±1.0	10.0±2.4	11.3±4.2	9.1±3.5	6.9±2.1	12.9±4.8	ns
		7D	4.1±0.4	4.9±0.5	11.3±2.5	5.6±2.3	6.5±0.7	21.2±11.7	*
	TIL ratios								
	CD4^+^/CD8^+^	3D	0.5±0.1	0.5±0.1	0.9±0.1	0.4±0.0	0.8±0.2	0.5±0.1	ns
		7D	2.7±0.6	3.7±0.5	3.6±0.8	1.4±0.5	1.1±0.1	1.0±0.7	˟ ˟
	CD8^+^/FoxP3^+^	3D	13.8±6.9	9.1±2.7	6.5±3.0	8.9±4.9	6.3±1.4	6.1±3.4	ns
		7D	6.3±0.4	5.7±0.2	3.4±0.7	10.6±3.6	12.3±1.5	7.8± 3.7	ns

Abbreviations: fr-GM-CSF: Free form GM-CSF, lip-GM-CSF: Liposomal GM-CSF, RFA: Radiofrequency ablation, BL: Blank Liposomes, TIL: Tumor infiltrating Lymphocytes, ns: no significant difference.

P value < 0.05.

* RFA-lip-GM-CSF vs all groups,

** RFA-lip-GM-CSF vs all groups, except RFA-BL,

****RFA-lip-GM-CSF, lip-GM-CSF alone, and fr-GM-CSF vs. RFA alone, RFA-BL, and sham,

˟ ˟ RFA-lip-GM-CSF, RFA-BL, RFA alone vs. lip-GM-CSF alone, fr-GM-CSF and sham.

**Fig 4:**
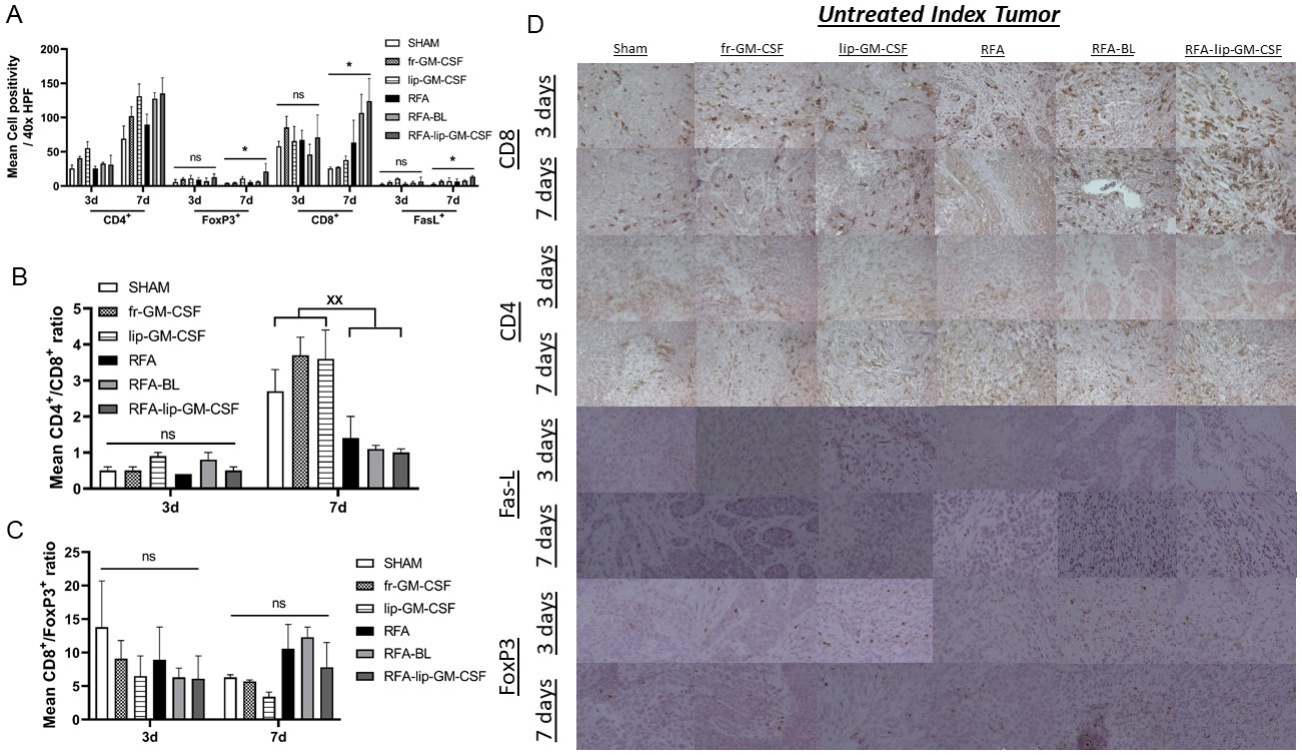
Effect of liposomal GM-CSF and RFA on tumor infiltrating lymphocytes in R3230 rat model on 3 and 7 days post ablation in the untreated index tumor. In the untreated index tumor (A), FoxP3^+^, CD8^+^, and FasL^+^ increased in RFA-lip-GM-CSF group compared to other groups at 7 days, but not at 3 days. CD4^+^/CD8^+^ ratio in all RFA treatment groups (RFA-lip-GM-CSF, RFA-BL, RFA alone) was lower than all non-RFA arms (lip-GM-CSF, fr-GM-CSF, and sham) at 7 days, but not at 3 days. For CD8^+^/FoxP3^+^ ratio, no differences in were noted in all groups at both 3 days and 7 days. Immunohistochemistry representative images of infiltrating TILs in the untreated index tumor (D). *, p< 0.05, RFA-lip-GM-CSF vs all groups, **, p< 0.05, RFA-lip-GM-CSF vs all groups, except RFA-BL, ˟˟, p< 0.05, RFA-lip-GM-CSF, RFA-BL, RFA alone vs. lip-GM-CSF alone, fr-GM-CSF and sham, ns, not significant.

In untreated index tumor, RFA-lip-GM-CSF increased CTLs at 7d compared to RFA-BL, RFA alone, lip-GM-CSF, fr-GM-CSF, and sham arms (p<0.05) (Fig 4A and 4D). Similarly, RFA-lip-GM-CSF increased FasL^+^ CTL activity at 7d compared to RFA-BL, RFA alone, lip-GM-CSF, fr-GM-CSF and sham arms (p<0.05). There were no differences in overall CTLs or CTL cytotoxicity in the untreated index tumor between treatment arms at 3d. Interestingly, all arms in which GM-CSF was administered (including RFA-lip-GM-CSF, lip-GM-CSF alone, and fr-GM-CSF alone) had increased Th1 cells in untreated index tumor at 3d compared to all non-GM-CSF arms (RFA alone, RFA-BL, and sham) (p<0.05). However, by 7days, significantly greater Th1 infiltration was observed in the RFA-lip-GM-CSF, RFA-BL, and lip-GM-CSF alone arms compared to RFA alone, fr-GM-CSF and sham treatments (p<0.05) (Fig 2B). Finally, FoxP3 IHC demonstrated a similar pattern of infiltration to CTLs. At 7 days post ablation there was significantly increased infiltration of T-regs in the untreated index tumor in the RFA-lip-GM-CSF arm in comparison to all other groups (p<0.05). At 3 days post ablation no significant infiltration patterns differences were detected in residual tumor (p>0.05) (Fig 4A and 4C) (Table 2).

### Combination therapy increases C8 positivity and shifts C4^+^/C8^+^ and CD8^+^/FoxP3^+^ ratios in the periablational zone

CD4/CD8 ratios demonstrated a shift towards CD8^+^ cells in the periablational zone with combined RFA-lip-GM-CSF compared to either RFA-BL or RFA alone at 7d (p<0.05) (Fig 3B). In the untreated index tumor, all RFA treatment groups (RFA-lip-GM-CSF, RFA-BL, RFA alone) demonstrated an equivalent shift towards CD8^+^ cells, as compared to no-shift in CD4/CD8 ratios for all non-RFA arms (lip-GM-CSF, fr-GM-CSF, and sham) (p<0.05). No significant differences in CD4/CD8 ratios were observed at 3d between any treatment groups (Fig 4B) (Table 2).

CD8/FoxP3 ratios demonstrated a shift towards CD8^+^ CTLs in the periablational zone following RFA-lip-GM-CSF at 7d compared to RFA-BL and RFA alone (p<0.05) (Fig 3C). This was not observed at 3d (p<0.05). Furthermore, no changes in the CD8/FoxP3 ratio were observed in untreated index tumor at 3d or 7d (Fig 4C) after ablation.

### Liposomes combined with thermal ablation induce a pro-immune cellular TME shift in synchronous distant tumor

**Fig 5:**
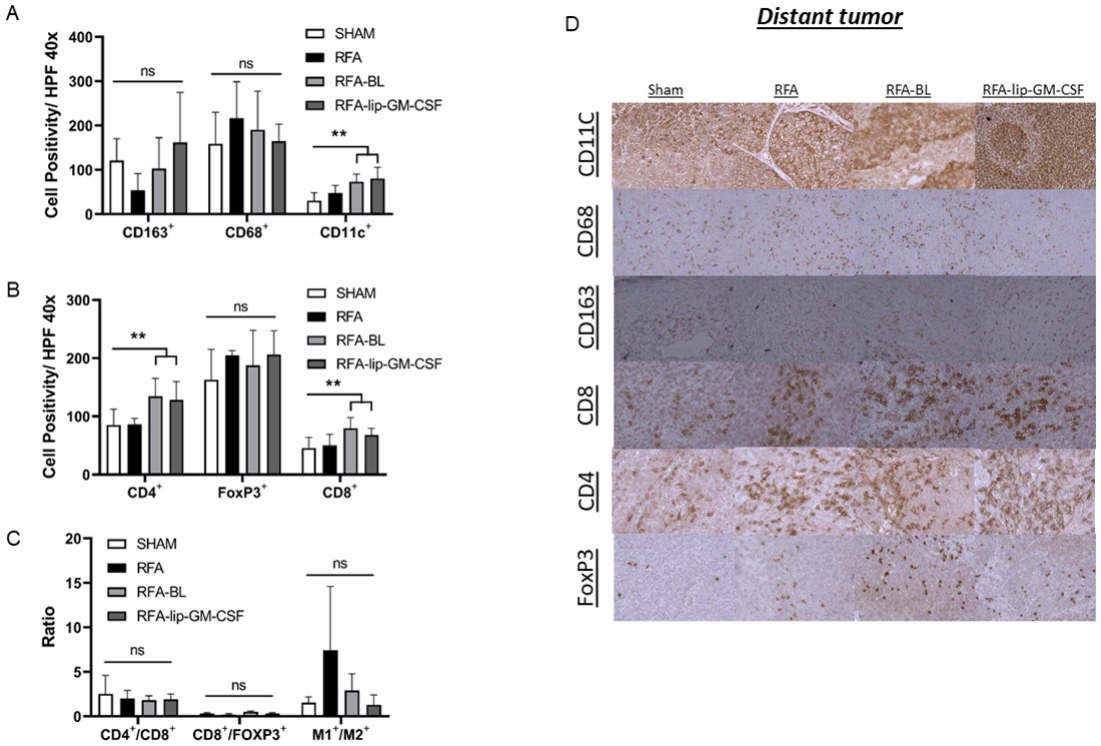
Effect of RFA and lip-GM-CSF on APCs and TILs infiltration in synchronous metastasis 7 days post ablation. (A) Expression of TAMs markers and DCs marker in a synchronous metastasis was examined 7 days post ablation of a primary tumor. CD11c^+^ expression, a DC marker, in RFA-lip-GM-CSF and RFA-BL group was higher than other groups, but CD163^+^ and CD68^+^ expression, markers for M2 and M1, showed no difference between groups. Expression of TILs markers in a synchronous metastasis (B) 7 days post ablation of a primary tumor demonstrated that CD4^+^ and CD8^+^ expression in RFA-lip-GM-CSF and RFA-BL group was higher than other groups, but FoxP3^+^ expression showed no difference between groups. All ratios, CD4^+^/CD8^+^, CD8^+^/FoxP3^+^, and M1^+^/M2^+^ did not demonstrate any differences between any of the treatment groups. Immunohistochemistry representative images of infiltrating APCs and TILs in synchronous metastasis. **, p< 0.05 RFA-lip-GM-CSF vs all groups, except RFA-BL, ns, not significant.

Both ablation treatment arms containing liposomal preparations, RFA-lip-GM-CSF and RFA-BL, had increased CD11C^+^ and CD8^+^ and CD4^+^ lymphocytes infiltration in the synchronous non-ablated tumor at 7d compared to either RFA alone or sham treatment (p<0.05) (Fig 5A–5C). No significant changes in CD68 and FoxP3 infiltration were observed (p>0.05) (Fig 5A–5C) (Table 3). Furthermore, RFA alone reduced CD163^+^ M2 macrophages in distant tumor compared to either RFA-lip-GM-CSF or sham arms (p<0.05, for all comparisons). No other differences were observed.

**Table 3:** Effect of combining thermal ablation and liposomal GM-CSF on distant tumor infiltration of tumor associated macrophages and dendritic cells, and tumor infiltrating lymphocytes.

*Distant tumor*			Sham	RFA	RFA-BL	RFA-lip-GM-CSF	P value
	Cellular infiltrates (PC/hpf±SD)						
	CD11c^+^	7D	30.3±17.9	47.7±17.1	73.0±17.2	80.5±25.0	**
	CD68^+^	7D	158.8±71.1	216.0±82.7	190.6±87.0	164.6±38.1	ns
	CD163^+^	7D	120.9±49.7	54.3±37.0	102.9±69.6	161.9±112.7	˟ ˟ ˟
	CD8^+^	7D	45.3±18.5	50.4±18.8	79.7±18.1	68.2±11.1	**
	CD4^+^	7D	85.0±27.0	86.0±10	134.0±31.0	128.0±31.0	**
	FoxP3^+^	7D	162.8±52.4	204.9±8.17	187.6±60.19	206.13±41.1	ns
	Cell Infiltrate ratios						
	M1:M2	7D	1.5±0.7	7.4±7.1	2.9±1.9	1.3±1.1	ns
	CD4^+^/CD8^+^	7D	2.5±2.1	2.0±0.9	1.8±0.5	1.9±0.6	ns
	CD8^+^/FoxP3^+^	7D	0.3±0.1	0.2±0.1	0.5±0.1	0.3±0.1	ns

Abbreviations: lip-GM-CSF: Liposomal GM-CSF, RFA: Radiofrequency ablation, BL: Blank Liposomes, ns: no significant difference.

P value < 0.05.

** RFA-lip-GM-CSF vs all groups, except RFA-BL,

˟ ˟ ˟ RFA alone vs. RFA-lip-GM-CSF and sham arms.

Calculated M1/M2, CD4^+^/CD8^+^ and CD8^+^/FoxP3 ratios demonstrated no significant differences between any of the treatment groups at 7day(p>0.05) (Fig 5C) (Table 3).

## Discussion

Percutaneous thermal ablation, a commonly utilized local therapy for treating focal cancers, can create an pro-immune local tumor microenvironment via hyperthermia-induced secondary reactions in its periablational rim, through release of tumor antigens, activation of pro-immune protein pathways (e.g. heat shock protein, interleukin pathways), and recruitment of inflammatory and immune cell populations to the treated tumor. Nevertheless, in the vast majority of cases, thermal ablation alone has been insufficient to activate clinically-meaningful anti-tumor immunity, and thus there has been increasing interest in augmenting ablation-induced anti-tumor pro-immune effects with adjuvant immunomodulation. Yet, a key barrier to currently reported combined drug-ablation approaches, either with direct intratumoral adjuvant drug injection or systemic administration, is inadequate delivery to the site of action (i.e. the periablational rim), and poorly matched kinetics as the drug does not peak within the time window required (i.e. 0-48h after ablation). Prior studies focused on combining thermal ablation with cytotoxic chemotherapy have often overcome these technical limitations using systemically-administered nanoparticles that: 1)preferentially deliver adjuvant drugs to the periablational margin following thermal ablation [36, 37], and 2) augment the local hyperthermia-induced tissue reactions by targeting specific mechanisms (such as cellular and protein-mediated activation) [28, 33, 38, 39]. Our current investigation builds upon these advances by using a nanocarrier approach to deliver a tumor microenvironment-modulating immunomodulatory agent to the periablational tissue at the time of thermal ablation.

In our study, GM-CSF was specifically selected as the ‘proof-of-concept’ immunomodulatory drug payload as it is activates maturation of antigen-presenting cell populations (macrophages and dendritic cells) in the setting of increased tumor antigen availability (both of which are present in the immediate post-ablation zone) [27, 40]. Indeed, our results demonstrate that adjuvant, liposomal GM-CSF administered at the time of thermal ablation successfully and specifically alters the tumor microenvironment cellular profile in the treated tumor after thermal ablation by increasing M1 macrophage recruitment. More importantly, this combination therapy also increases downstream 7-day activated (Fas-L^+^) CTL recruitment and TIL ratios of up to 5-fold within the periablational rim, underscoring the ability of upstream and early immune stimulation to activate later stages in anti-tumor immunity in this clinical setting. Furthermore, RFA-lip-GM-CSF also enhanced the immune microenvironment in the treated tumor beyond the periablational rim, demonstrating that this immune upregulation is not only a by-product of the partial injury and hypervascularity observed in the periablational rim [41], but represents a more global change induced throughout the index tumor. For example, changes in multiple pro-immune cell populations were observed at 7d in the index tumor a substantial distance from the periablational rim, including increased DCs (CD11c), Th1 (CD4^+^) and CTL infiltration (CD8^+^) and CTL activity (Fas-L^+^). These findings confirm the ability of adjuvant nanodrugs to modulate the local periablational tumor microenvironment to activate early and late stages of anti-tumor immunity throughout the target tumor.

Second, our study confirms that a combination ablation / nanodrug approach with immunostimulation can also activate downstream anti-tumor immunity in distant non-ablated tumors. In the double tumor model, which simulates a scenario of synchronous metastasis, increased DCs and TILs (CTL and Th1) infiltration was observed in RFA-lip-GM-CSF and RFA-BL groups when compared to RFA alone or sham controls. Interestingly, macrophages and T-regs levels were similar to sham. Furthermore, infiltrating cells, primarily CTLs and TILs, rely on antigen cross presentation to tumor neoantigens to infiltrate distant tumors. By contrast, macrophage infiltration relies primarily on inflammatory signals to infiltrate tumors. This may suggest a tumor specific anti-tumor response elicited by combining ablation with a liposomal carrier, irrespective of agent. Regardless, these results underscore the role for targeted drug delivery to the periablational rim as a potential method to activate a tumor-specific systemic immune response.

Third, our results confirm that the liposome nanocarrier platform itself (i.e., the combined RFA and blank liposome arm) also can induce effects in the local tumor microenvironment. In this study, the RFA-BL treatment also resulted in increased cellular trafficking, though often to a lesser effect than RFA/liposomal GM-CSF. Prior studies have also shown that the lipid carrier can introduce some effects including inflammation and cytotoxicity, and that the carrier can be altered (by changing the lipid components) to increase this effect [34, 42, 43]. Further future optimization may be possible with the potential use of alternative carriers that have variable sizes and circulation times to modify the drug delivery profile. Alternatively, thermally-sensitive carriers that have more rapid drug release profiles may prove useful for this task [44]. Ultimately, optimization of the carrier platform to align its properties to the intended target mechanism to maximize immunomodulation, is likely to be beneficial.

Fourth, this study underscores the temporal nature of the tumor microenvironment cellular composition following tumor ablation alone or combination treatment arms. For instance, in the periablational rim, M2 macrophages demonstrated early activation followed by normalization, however, CTL infiltration persisted into later time points. This underscores the need to sample / surveil at the correct time for downstream effects when differentiating degree of treatment response. Indeed, the dynamic nature of the post-ablation local tumor microenvironment and systemic immune milieu will impact how future combined local and systemic strategies may need to be combined. Recent studies have used dynamic contrast MRI with gadolinium-labelled antibodies to markers of immune cell populations may be helpful in providing a non-invasive method of tracking the tumor immune microenvironment following RFA-lip-GM-CSF in future studies [45, 46].

We acknowledge that not all cellular activation from adjuvant lip-GM-CSF can be considered favorable. For example, we note that combining RFA with liposomal GM-CSF also recruited some cell populations known to promote immunosuppression and tumor progression. For instance, in the periablational rim and untreated index tumor, M2 macrophages and T-reg lymphocytes (T-reg) increased following thermal ablation. However, this did not result in a downstream affect in synchronous metastasis, where no increase in undesirable APCs or TIL sub-populations was observed. This might indicate that the adjuvant GM-CSF-induced macrophage activation was indiscriminate, and included both M1 and M2-type macrophage. Indeed, the M2:M1 shift (observed at 3 days after thermal ablation) is generally considered pro-oncogenic and immunosuppressive. This underscores the nuanced balance, between activation of inflammation versus anti-tumor immune pathways previously observed following thermal ablation [30, 40]. Regardless, these undesirable findings may be explained by a number of mechanisms. First, GM-CSF is a potent stimulator of APCs including M2-type macrophages [31]. Additionally, GM-CSF promotes MDSCs, which are also potent immunosuppressive cell populations that enhance polarization of TILs into T-regs [47]. Furthermore, DCs are known to play a role in immunosuppression through mediation of M2 macrophage promotion [48]. As such, in future studies, immunostimulating agents that have more specific actions, that hone specific ablation-induced immune response, may need to be explored.

Ultimately, our study also raises several additional further questions that call for the need of additional lines of inquiry. First, the optimal single or combination of drugs to be delivered to the local ablation zone to maximize anti-tumor immune activation remains to be determined. This may include drugs targeting different mechanisms (such as immune stimulation with release of immune suppression, akin to the ‘press the accelerator and release the brake’ model), and will likely need to be ‘personalized’ to a given tumor immune micronevironment phenotype. Second, as noted above, optimal nanocarrier characteristics still need to be considered, specifically with regard to maximal delivery in the immediate post-ablation period, optimizing local payload drug release, and enhancing active effects of the carrier itself. Finally, determining ideal drug dosing strategies (e.g. single or multiple dosing) including kinetics and timing with ablative therapy and how local therapy can be combined with systemic agents (e.g., checkpoint inhibitors) needs to be explored.

Several limitations of this study ought to be mentioned. First, this proof-of-concept study was performed in a single animal tumor model, which was selected as a well-studied immunocompetent tumor microenvironment model for thermal ablation and liposomal drug delivery. Thus, additional validation in other representative models of cancer type, organ location, and tumor immune phenotype is required. This study also focuses on the cellular composition as a single aspect of the tumor immune environment, and additional studies on activation of protein pathways would be beneficial. Additionally, partial incomplete treatment of the index tumor was purposefully performed in these studies to assess the effect of adjuvant immunomodulation on the tumor microenvironment. However, in current clinical practice, local complete ablation (including achieving an ‘ablative margin’) is the most commonly used treatment strategy and technical endpoint. Thus further studies are necessary in models of complete ablation to study the influence of adjuvant local immunomodulation. Finally, this study employed one type of thermal ablation (i.e., radiofrequency ablation), and additional validation in other methods of local tumor ablation will be required.

In conclusion, adjuvant systemically administered liposomal GM-CSF can be successfully used to induce a pro-immune cellular shift in the local tumor microenvironment following local thermal ablation, with early downstream changes in distant untreated tumors as well. Further studies are required to validate our findings such as improved small animal survival and decreased recurrence rates.

## Supporting information

S1 File Supplemental material including tumor growth measurements, tumor harvesting and immunohistochemistry antibody combinations are available as a separate file online. (DOCX)

## Abbreviations

APCs: antigen presenting cells
CTL: Cytotoxic T- Lymphocyte
DC: Dendritic cells
GM-CSF: granulocyte-macrophage colony stimulation factor
MWA: Microwave ablation
RFA: Radiofrequency ablation
TAMs: Tumor associated macrophages
Th-1: T-helper lymphocytes-1
TILs: Tumor infiltration lymphocytes
T-reg: Regulatory T-lymphocytes
