# Supplementary material for: Combined thermal ablation and liposomal granulocyte-macrophage colony stimulation factor increases immune cell trafficking in a small animal tumor model
Source: PLoS One. 2023 Oct 26;18(10):e0293141. doi: 10.1371/journal.pone.0293141 (PMC10602257; doi:10.1371/journal.pone.0293141)
Supplement: S1 File — (DOCX) [file pone.0293141.s001.docx]

**SUPPLEMENTAL MATERIALS**

**Tumor Growth Measurements:** The average diameter of tumor was recorded by measuring in both longitudinal and transverse diameters using mechanical calipers. Tumors were measured every alternative day until reached 6-7mm, from that point they were measured every day. The rats were incorporated in the study when tumor size was in a range of 12-14mm in diameter, at that time animals were divided into four groups according to protocol where each group consisted of 5 animals at least. The animals were randomized in such a way that the mean starting tumor size was almost similar with no statistical difference in all comparative treatment groups. After RF ablation/sham treatment and lip-GM-CSF/placebo administration, tumor measurements were kept obtaining for seven additional days, once in a day.

**Tumor Harvesting:** Animals were sacrificed at 3d and 7d of RF ablation/sham treatment for tissue harvesting. The harvested tumors were sectioned perpendicularly to the direction of electrode insertion. The half of the tumor containing the central section was placed in a 10% formalin container for overnight at 4°C for fixation, then embedded in paraffin, and sectioned at a thickness of 5μm for histological assessment. Tissues were stained with hematoxylin eosin for histopathology.

| Table S1: Primary and secondary antibody combinations used in phase I and II Immunohistochemistry assays | | | | | | | |
| --- | --- | --- | --- | --- | --- | --- | --- |
| Primary Antibody | Manufacturer | Catalog no. | Concentration | Secondary Antibody | Manufacturer | Catalog no. | Concentration |
| CD68 | Abcam | ab31630 | 1:1000 | Anti mouse | Abcam | ab205719 | 1:2000 |
| CD163 | Santa cruz | sc-58965 | 1:50 | Anti mouse | Santa Cruz | sc525408 | 1:100 |
| CD4 | Abcam | ab237722 | 1:2000 | Anti Rabbit | Abcam | ab209101 | Ready to use |
| CD8 | Santa Cruz | sc-70802 | 1:100 | Anti mouse | Santa Cruz | sc-525408 | 1:25 |
| FoxP3 | Thermo Fisher | 14-5773-82 | 1:50 | Anti mouse | Abcam | ab205719 | 1:2000 |
| Fas-L | Santa Cruz | sc19988 | 1:50 | Anti mouse | Abcam | ab205719 | 1:2000 |
| CD11C | biorbyt | orb13554 | 1:500 | Anti Rabbit | Abcam | ab209101 | Ready to use |
